# Listen to the outpatient: qualitative explanatory study on medical students’ recognition of outpatients’ narratives in combined ambulatory clerkship and peer role-play

**DOI:** 10.1186/s12909-018-1336-6

**Published:** 2018-10-03

**Authors:** Noriyuki Takahashi, Muneyoshi Aomatsu, Takuya Saiki, Takashi Otani, Nobutaro Ban

**Affiliations:** 1 Department of General Medicine / Family and Community Medicine, Nagoya University Graduate School of Medicine, 65 Tsurumai-cho, Showa-ku, Nagoya, 466-8550 Japan; 2 Department of Education for Community Oriented Medicine, Nagoya University Graduate School of Medicine, 65 Tsurumai-cho, Showa-ku, Nagoya, 466-8550 Japan; 3 Department of General Medicine, Nagoya University Hospital, 65 Tsurumai-cho, Showa-ku, Nagoya, 466-8560 Japan; 4 Department of Medical Education, Saku Central Hospital, 197 Usuda, Saku, 384-0301 Japan; 5 Medical Education Development Center, Gifu University, 1-1 Yanagido, Gifu, 501-1193 Japan; 6 Department of Educational Sciences, Graduate School of Education and Human Development, Nagoya University, Furo-cho Chikusa-ku, Nagoya, 464-8601 Japan; 7 Medical Education Center, Aichi Medical University, 1-1 Yazakokarimata, Nagakute, 480-1195 Japan

**Keywords:** Ambulatory clerkship, Patients’ narratives, Medical interview, Outpatient, Peer role-play, Undergraduate medical education

## Abstract

**Background:**

Understanding patients’ narratives has been associated with methods of improving care that go beyond what may be regarded as a “narrow” view of scientific medicine. Medical interview training in which medical students develop understanding of the importance of patients’ narratives is receiving increased attention. However, students generally receive education on patients’ narratives that does not distinguish inpatients and outpatients. No studies exploring the characteristics of outpatients’ narratives have been reported. We developed an educational program combining ambulatory clerkship and peer role-play using actual narratives from outpatients that students had encountered during their clerkship. These narratives were used as peer role-play scenarios in which the students acted as outpatients. This study explored what and how medical students learned about the characteristics of outpatients’ narratives through this original educational program.

**Methods:**

Participants were 70 fifth-year medical students from Nagoya University, Japan. We conducted 13 focus groups, based on a convenience sample of 11 groups in 2012, one group in 2013, and one group in 2017 (from 17 clinical groups in each year). Focus group transcripts were analyzed using the “Steps for Coding and Theorization” qualitative data analysis method. We assessed medical anthropological findings regarding narratives in a conceptual framework.

**Results:**

Patients’ narratives as perceived by medical students were divided into four quadrants by two axes: medical versus lived content, and objective versus subjective structure. Students recognized that outpatients’ narratives mainly used a subjective structure, but were mixed and crossed each quadrant. This was described as “irreproducibility.” Students also recognized that narratives of simulated patients and inpatients were mainly limited to a medical-lived content with an objective structure. These differences in narrative characteristics were recognized through students’ previous interactions with simulated patients and inpatients.

**Conclusions:**

Despite some limitations, medical students learn about patients’ narratives in our original educational program in a way that would be difficult to achieve through training using simulated patients or inpatients.

## Background

Doctor-patient communication is at the heart of the practice of medicine. However, communication frequently goes awry. Doctors’ consideration of patients’ narratives is essential to improve patient care and foster empathetic attitudes and patient-centered care [1–3]. Various educational programs to develop medical students’ understanding of patients’ narratives have been reported [3–8]. Understanding these narratives is a key purpose of medical interview training [9]. Therefore, it is important to design learning strategies for undergraduate medical education that incorporate patients’ narratives in medical interview training. In 2001, we developed an educational program that combined outpatient encounters and medical interview training using peer role-play [10, 11]. In this program, actual narratives from outpatients that students had encountered during their ambulatory clerkship were used as peer role-play scenarios in which the students acted as outpatients. This aimed to resolve the “problematic reality” of peer role-play scenarios. In our role-play exercises, we used to use scenarios written by medical students based on their own experience as a patient or a patient’s family member [10].

To date, characteristics of patients’ narratives have mostly been described without distinguishing inpatients and outpatients [12–15]. Therefore, although several studies have reported medical students’ recognition of real patients’ narratives [16, 17], no research has explored differences in the characteristics of inpatient and outpatient narratives as perceived by medical students. Ambulatory or outpatient training provides a good opportunity for students to experience medical interviews and understand patients’ narratives [18, 19]. In ambulatory practice, more attention needs to be directed to patients’ narratives than in hospitalized practice to ensure better patient management [18, 20]. Therefore, it is important to explore how medical students understand real patients’ narratives in ambulatory settings. We are empirically convinced that this original educational program combining outpatient encounters and medical interview training using peer role-play supports students’ acquisition of significant learning about these narratives that is not found elsewhere.

### Study aims

This study was guided by two research aims:

1. to explore the characteristics of outpatients’ narratives that medical students learn to identify; and
2. to explore how students learn about these narratives in this original educational program.

## Methods

### Setting

#### Pre-clerkship course

**Fig. 1:**
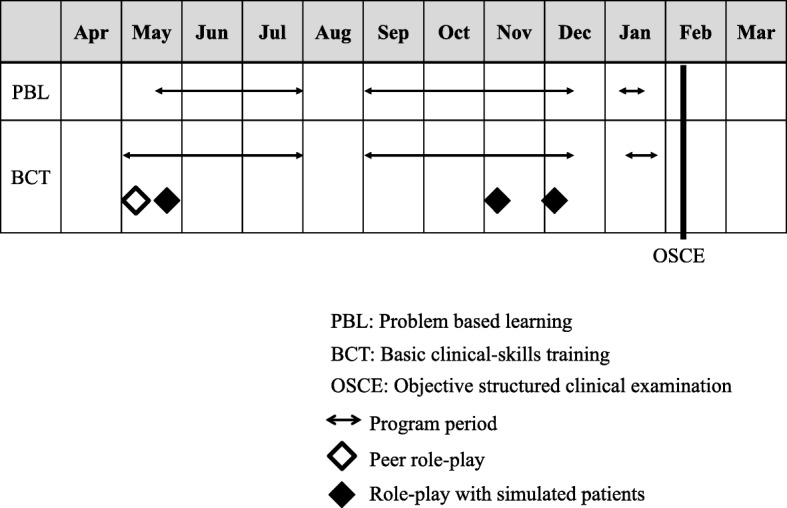
Curriculum for fourth-year students at Nagoya University School of Medicine

Each grade in Nagoya University School of Medicine has around 107 students. As with other medical schools in Japan, our university offers a 6-year curriculum, with the final 2 years generally spent in clinical clerkships [21]. Fourth-year students, who are in the last year of the pre-clerkship course, receive problem-based learning [21] and basic clinical-skills training [22] (Fig. 1).

Basic clinical-skills training introduces students to medical interviews through participation in a peer role-play session and three additional sessions of interview practice with simulated patients (SPs). Using prepared scenarios, students perform the peer role-play in groups of three, and take turns assuming the roles of patient, doctor, and observer. Feedback is provided according to a prepared assessment sheet. Following the peer role-play, all students participate in small group (10 groups in total) SP sessions facilitated by department faculty. Students take turns assuming the doctor role in 7-min mock interviews, and receive feedback from their peers and the SP about their communication skills, again based on a prepared assessment sheet.

#### Clinical clerkship in the Department of General Medicine

**Fig. 2:**
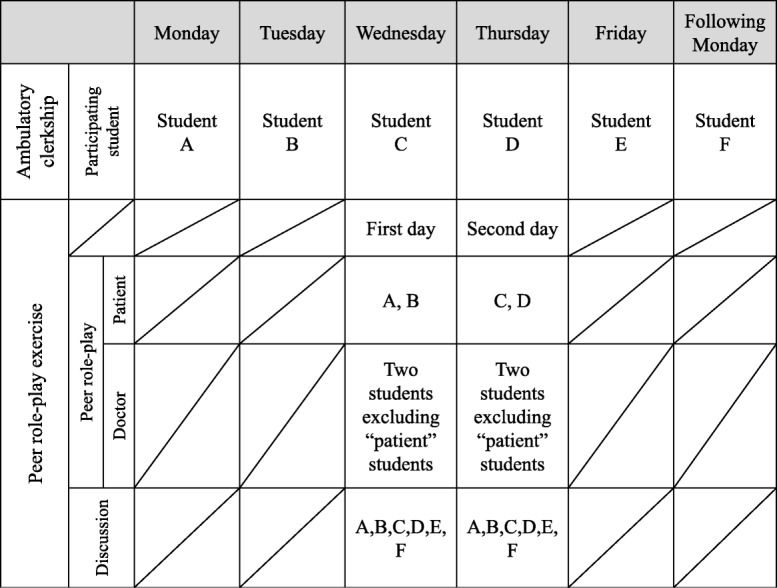
Weekly schedule and participation of students in the ambulatory clerkship and peer role-play exercises

The clinical clerkship for fifth-year medical students has a fixed curriculum comprising 1- to 2-week terms (22 terms in total). During this time, students rotate through 37 clinical departments in the university hospital in groups of five to seven. These clerkships include medical interviews with inpatients and outpatients. Six days are spent in the Department of General Medicine, which offers several educational programs. One of these programs is the ambulatory clerkship with peer role-play, which is part of our program [10, 11] (Fig. 2).

#### Ambulatory clerkship

The ambulatory clerkship is facilitated by the two of the present authors (MA and NB) and other faculty members. Each student who rotates through the clerkship has an opportunity to interview a patient once during the weekday morning ambulatory clinic. Before the student interview, the attending doctor in charge of the ambulatory clinic on that day identifies a patient who is willing to be interviewed by a medical student. The student is then allowed 30 min to interview that patient. After the interview, the student invites the patient to the attending doctor’s examining room, and presents the case to the attending doctor in front of the patient. Next, the attending doctor obtains additional information and performs a physical examination while the student observes. After the patient encounter is completed, the student and the attending doctor discuss the case, and the student summarizes the patient’s profile for the subsequent role-play.

#### Peer role-play exercise

Peer role-play sessions are held at the clinical simulation center. Students act patient roles based on narratives that they attended in their ambulatory clerkship. This session includes five to seven students and one supervising teacher. The patient does not participate. Before beginning the role-play, all students in the clerkship group assemble in the debriefing room and the facilitator reviews the objectives of the role-play and group discussion. Then, two simultaneous peer role-plays are performed in simulated consultation rooms that are observable via video monitors in the debriefing room. The role-play and discussion are supervised by one of the present authors (NT) and a junior staff member.

In the role-plays, two students who previously interviewed patients during the clerkship assume the roles of their respective patients and two other students play doctor roles. The remaining students and supervising teachers observe the role-play in the debriefing room. The students are allowed 10 min to complete their interview, and each role-play is videoed. Role-play participants are given specific tasks, such as:

1. The student in the doctor role attempts to use acquired knowledge about communication skills, apply clinical reasoning, and probe for the patient’s perspective.
2. The student in the patient role discloses the information shared at the ambulatory clinic and makes ad-lib responses when asked for unknown information. The student “patient” may disclose the patient’s true age, sex, chief compliant, and a fictitious name.
3. During the interviews, student observers wait in the debriefing room. They can watch the role-play on the monitors but are unable to hear the live conversation. This is intended to keep their interest until the time to watch the role-play video as a group.

After the role-play exercise, all students assemble in the debriefing room for a structured group discussion. First, the student who played the doctor role briefly reflects on the communication skills used during the interview. Then, all group members watch the role-play video (with audio), during which the student “doctor” provides detailed comments about specific communication skills demonstrated. Next, all group members are invited to discuss the use of communication skills in the simulated encounter.

In a second activity, the student “doctor” summarizes the information obtained in the interview, and the students are invited to apply their own clinical reasoning to the case. In the discussion that follows, the student “doctor” and student observers have further opportunity to probe the student “patient” regarding their medical history to improve their reasoning. Finally, the student “patient” discloses the information actually gathered from the patient during the visit with the attending doctor. To encourage student-centered learning, the facilitator does not take an active part in the discussion and does not interrupt unless the discussion strays from the aim of the session [10]. At the end of the session, the facilitator takes questions about communication and clinical reasoning, and finally summarizes the session. The second (simultaneous) group session is conducted in the same way.

### Data collection

Data were collected through focus groups by the author (NT) who facilitated the peer role-play exercise (peer role-play and discussion) on a Wednesday, after finishing the formal educational program for the day. Convenience sampling was used with 11 groups in 2012, one group in 2013, and one group in 2017, which were selected from 17 groups in each year. The 2013 and 2017 groups were selected because of the need to strengthen the theoretical construction. Convenience sampling in this case referred to inviting students to participate in this research when it was necessary to perform additional data sampling after analyzing previous data.

Focus groups were chosen as the data collection method after determining that homogeneous focus groups (i.e., in terms of age, sex, race, and social background) would not be greatly compromised by the power imbalance between the researcher and participants [23]. In the focus group sessions, students were first asked the open-ended question “How do you feel about this program?” Based on students’ responses, the author/researcher (NT) encouraged discussion among the group and ensured that all participants had sufficient opportunity to express their views. Additional questions were asked about students’ recognition of patients, such as “Do you think you can understand patients’ perspectives (illness)?” and “Why do you think so?” Based on emerging findings, the questions posed in focus group discussions changed over time to more deeply explore students’ understanding. Each focus group lasted 40–60 min.

### Data analysis

Recordings of focus group discussions were transcribed verbatim immediately after the interviews. Transcripts were analyzed using the “Steps for Coding and Theorization” method, which is a four-step coding process used to identify themes and constructs, develop a story-line by weaving the themes and constructs, and finally offer theories [24–30]. We chose this approach because of its explicit analytic process, and validation of theory by assuring opportunity to show critiques and falsifiability.

### Conceptual framework

We used narratives as the conceptual framework. The purpose of a conceptual framework is to clarify the nature of a problem and help to develop the research question and design [31]. We used narratives to clarify our research question and avoid insufficiency and confusion in the analysis caused by any vagueness of the wording used.

#### Concepts of narratives

Concepts relating to narratives may be described by medical anthropology. A narrative refers to “the imaginative linking of experiences and events into a meaningful story or plot” [32]. “Patient” was described as the suffering person [12]. “Patient perspective” was considered as “illness” [33, 34] and described as “the innately human experience of symptoms and suffering” [12]. In contrast to illness, disease was described as the problem from the practitioner’s perspective, which was reconfigured as an alteration in biological structure or functioning [12].

## Results

In total, 13 focus groups were conducted. Of the 70 participating students, 58 (42 men, 16 women) were from a class of 108 fifth-year students in the 2012 school year, five (men) were from the class of 107 fifth-year students in the 2013 school year, and five (men) were from the class of 113 fifth-year students in the 2017 school year. The 2013 and 2017 focus groups were conducted because of the need to strengthen the theoretical construction as a result of previous analysis. We constructed and generated theoretical writing by analyzing the results of these focus groups, and explored medical students’ recognition of characteristics of outpatients’ narratives. We also investigated how students’ recognized characteristics of outpatients’ narratives through the original educational program. This program combined outpatient encounters and subsequent medical interview training using outpatients’ narratives as scenarios in peer role-play exercises.

### Medical students’ recognition of characteristics of patients’ narratives (two axes)

**Fig. 3:**
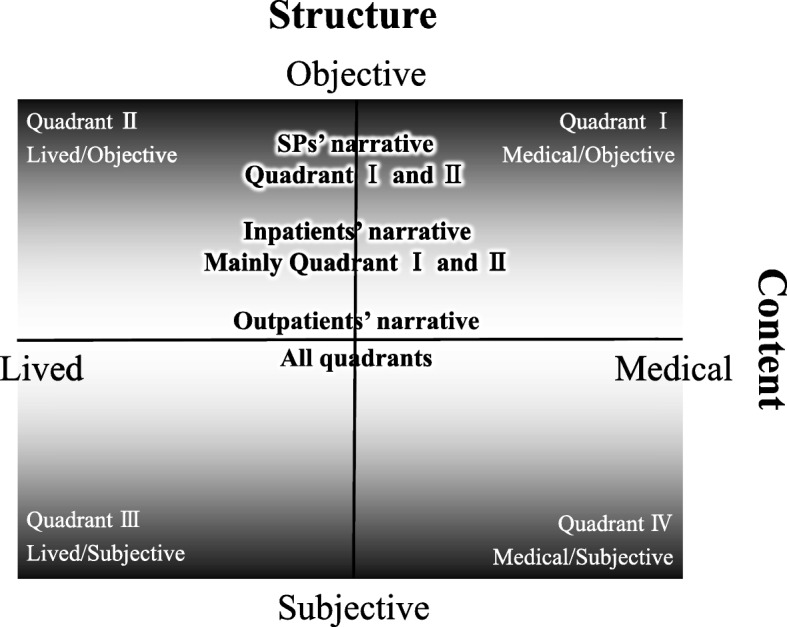
Characteristics of patients’ narratives on two axes. The content axis reflects whether the content was medical or lived, whereas the structure axis has degrees. Objective and subjective structure become stronger moving toward the edges; nearer the center sit more poorly-arranged narratives, which had neither objective nor subjective structures

Outpatients’ narratives as conceived by medical students were classified using a two-by-two grid of four quadrants by two axes based on “content” and “structure” (Fig. 3). Content represented the information contained in patients’ narratives, and structure represented the systems that patients’ narratives contained. Content was determined on an axis between “medical” versus “lived,” and structure on an axis between “objective” versus “subjective.” Medical students conceived these characteristics based on contrasts with previous interactions with SPs in their pre-clinical course and inpatients encountered in clinical clerkships in other departments.

### Content: Medical versus lived

Medical students recognized that this axis represented whether the content of the narrative was medical or lived; the axis had no “center.” Medicine is described anthropologically “as a cultural system, a system of symbolic meanings anchored in particular arrangements of social institutions and patterns of interpersonal interactions,” in which patients and healers are basic components [35].

### Lived content

#### Lived content compared with SPs’ narratives

Medical students compared outpatients’ narratives with those of SPs. As SP scenarios were usually based on medical knowledge, students recognized that medical content and sometimes lived content in SPs’ narratives were relatively well organized.

*SPs’ speech is well organized; so I can summarize not only the present illness, but also their life.* *(Doctor role, woman, Group 5; 132)*

*SPs’ scenario is better prepared than outpatients’; I realize it by asking them carefully.* *(Doctor role, man, Group 12; 54)*

Conversely, participating students perceived that outpatients sometimes gave priority to narrating lived content and their feelings.

*But the outpatient talked about a wedding ceremony that seemed unrelated to the current condition, for example.* *(Patient role, woman, Group 1; 23)*

*For another example, (the outpatient said) how their research at university went (…), he felt busy because he woke up at 9 o’clock and kept doing an experiment until 6 o’clock at night, something like that.* *(Patient role, man, Group 3; 101, 166)*

*(The outpatient) is single and jobless, and fell victim in Tokyo and was scared by the earthquake. He distrusted people in Nagoya because they didn’t understand the earthquake because Nagoya didn’t experience the earthquake strongly.* *(Patient role, man, Group 8; 68, 72)*

Lived content was considered non-medical content, consistent with Kleinman’s view that patients “perceive, and live with (…) symptoms and disability” [12]; patients live in a life-world as a living person. Life was described from a medical anthropological perspective as a “lived experience” [32]. In other words, outpatients recognized illness as a part of their life. Therefore, it was natural for them to give priority to disclosing non-medical or “lived content.” [36].

#### Lived content compared with inpatients’ narratives

Medical students identified that outpatients’ narratives did not voluntarily include medical content, which is usually included in inpatients’ narratives without being asked.

*I think inpatients might understand roughly what to tell us through previous questions by doctors (or other health professionals). But this time, I failed to catch information from outpatients. It is quite different.* *(Doctor role, man, Group 6; 159)*

The students recognized that inpatients had already received explanations from doctors about their disease during hospitalization. Furthermore, they considered that inpatients gained knowledge about their disease through self-learning and were therefore able to narrate in terms of medical content.

*When patients go to hospital, they are told by doctors about the disease they are suffering from and what kinds of therapeutic plans they should receive.* *(Observer, man, Group 13; 114)*

*Many inpatients look for their diseases by themselves.* *(Patient role, man, Group 13; 109)*

Participating students also perceived that inpatients’ narratives also contained lived contents.

*(The inpatient) is a police detective; he told me about an episode of bravery.* *(Observer, man, Group 13; 89)*

#### Structure: Objective versus subjective

Medical students recognized that narrative structure was determined on an axis between objective and subjective, which was consistent with Popper’s description [37]. Objective was based on “objective knowledge,” when “sufficient reason” had been demonstrated for distinguishing “true and certain knowledge” from mere opinion or belief. Subjective was based on “subjective knowledge,” which was a disposition to become conscious in the form of a belief, opinion, or state of mind. Narratives tended to be scientific if the objective structure was stronger and non-scientific if the subjective structure was stronger. However, systematic objective and subjective structures were both possible if narratives had strong structures.

#### Objective structure

##### Objective structure compared with SPs’ narratives

SPs are trained to narrate prepared fixed scenarios [33], or “certain knowledge” to clearly differentiate them from SPs’ opinion or belief. Therefore, SPs’ narratives were considered as having strong objective structures. Students recognized these characteristics of SPs’ narratives, which were systematically placed in each category, such as time series of episodes and current and past medical history.

*Most SP narratives are typical cases. No much ambiguity is contained in them.* *(Patient role, man, Group 12; 105)*

*For example, if I asked SPs “Do you want to say anything else?” they would disclose their concern about the relationship between disease and stress because they knew [about] the relationship.* *(Patient role, man, Group 12; 178)*

However, the students recognized that sometimes outpatients’ narratives also contained an objective structure.

*I asked and (the outpatient answered) “I had a stinging pain 2 or 3 weeks ago, and it had changed like this, and recently disappeared.”* *(Patient role, man, Group 2; 70)*

*Yesterday (in ambulatory practice), I thought about the patients’ understanding what she was the most worried about and what she needed us to do for her. I’m sure anyone would be surprised to see bloody urine. The outpatient’s complaints were never inconsistent. Basically, I’m sure she wouldn’t start talking about something like that suddenly for no reason.* *(Patient role, woman, Group 4; 181, 197, 199)*

##### Objective structure compared with inpatients’ narratives

Medical students perceived that inpatients’ narratives mainly had an objective structure, and outpatients’ narratives had a less objective structure than inpatients.

*Student Te (Doctor role, man): I had talked with inpatients on many occasions. My impression was that talking with an outpatient was quite different. Inpatients had already organized their thoughts by themselves…*

*Student Ta (Observer, woman): Probably they gave the same history repeatedly,*

*Student Te: Their answers were usually quite smooth.*

*Researcher: Does “smooth” mean that inpatients answered open questions coherently?*

*Student C (Patient role, woman): When I asked inpatients about their progress so far, they answered with not much interruption how they went to a previous hospital, what kinds of examinations they received, and what they were told (by doctors)…* *(Group 6; 143–148)*

*In the case of inpatients, they answer my questions easily without my needing to make a particular effort.*

*(Doctor role, man, Group 10; 96)*

*Student K (Patient role, man): (Inpatient) looks like experienced,*

*Student Te (Doctor role, man): Along with time series,*

*Student Ta (Observer, woman): They talk fluently.* *(Group 6; 153–155)*

The students recognized that inpatients’ narratives seemed to acquire objective structure because they were repeatedly asked about their condition by medical staff and medical students.

*Some inpatients are assigned students many times.* *(Observer, man, Group 13; 95).*

#### Subjective structure

##### Subjective structure compared with SPs’ narratives

Medical students recognized that some outpatients’ narratives had a strong subjective structure. However, although such narratives had strong subjective structures, they also had causal relationships of their own. Therefore, outpatients’ narratives were sometimes one-sided. Students recognized this characteristic of outpatients’ narratives through comparisons with SPs’ narratives. In addition, most subjectively structured outpatients’ narratives had lived content.

*Student I (Doctor role, man): It would be more realistic if the outpatient said something that she or he wanted to talk to the doctors about.*

*Student O (Patient role, woman): But the outpatient kept talking about a wedding ceremony that was held around the time when he went to a previous hospital. Though he remembered urological and heart diseases, he didn’t remember what operations he received. So I can’t understand his medical history only by what he said.* *(Group 1; 22–23)*

*Suddenly, an incoherent topic came up (in outpatient narratives).* *(Doctor role, man, Group 5; 381)*

*(Outpatient interaction) is not expected communication. For example, whenever I asked an outpatient, the patient rarely gave me a coherent answer like the one based on a scenario. I realize that I would be puzzled by outpatients’ topics going without my expectation.* *(Observer, woman, Group 7; 446, 450)*

However, the students recognized that subjectively structured outpatients’ narratives sometimes also contained medical content.

I think they (outpatients) come to a hospital with which they want to consult. However, patients’ answers are not always what we expect. For example, when I asked a patient about past illness, his answers were about things that were beside the point, such as “I had low back pain, and I went to the G hospital…Nerve block injection was really good for me, and I wanted to have an additional injection to the cervical nerve, but the doctor did not give me the shot…”(Patient role, woman, Group 7; 60, 62)

The students recognized outpatients’ narratives as objectively incoherent compared with SPs’ narratives.

*Student A (Patient role, man): I can’t stop thinking about the answers of SPs’ scenarios. (When talking with SPs), I feel like I’m heading for something.*

*Student F (Observer, woman): Me too. When I interview SPs, I tend to try to find where hints are hidden.*

*Student U (Observer, man): If the SP said something like key words, it must be that particular disease!*

*Student F: But outpatients have various key words. I can’t focus on particular disease. I have to consider not only the one word but also the other words, which are possibly related to the current condition. Today’s outpatient’s narratives (which were shared in the peer role-play exercise) were very broad and difficult to summarize for me.* *(Group 1; 59–65).*

However, they recognized outpatients’ narratives as subjectively coherent.

*Outpatients feel completely different things from doctors. For example, to succeed in patient management, we have to understand the patient’s thoughts such as, “Punching walls makes me suffer from rheumatoid arthritis.”* *(Observer, man, Group 8; 83, 97)*

##### Subjective structure compared with inpatients’ narratives

Medical students recognized that inpatients’ narratives had an objective structure because they knew their diagnosis and therapeutic plan. However, outpatients’ narratives had a subjective structure because a diagnosis and therapeutic plan had not been established at the time of the students’ encounters. The students perceived these differences by comparing inpatients’ and outpatients’ narratives.

*Student H (Doctor role, man): I feel (it is) more difficult to approach outpatients than inpatients. I can’t guess what outpatients will say.*

*Student W (Observer, man): I think outpatients don’t have a definite diagnosis when they visit an ambulatory clinic. And also, they don’t grasp their medical conditions. But inpatients roughly know their therapeutic plan to manage their symptoms.* *(Group 13; 80, 81)*

The students also recognized inpatients’ narratives as objectively coherent.

*Inpatients understand their medical conditions because they have already been explained by their doctors. So, they tell us coherently about the purpose of hospitalization. Outpatients, however, they don’t know why they’ve come to the hospital. They just complain of pain or something like that.* *(Observer, man, Group 13; 101)*

Conversely, outpatients’ narratives were recognized as subjectively coherent.

*(Outpatients) enthusiastically complained that the size was like a mouse. By talking, I understand the patient’s concerns, symptoms, and what the patient wanted to say.* *(Observer, man, Group 13; 18)*

#### Non-subjective, non-objective structure.

Medical students recognized that outpatients’ narratives sometimes had a non-subjective, non-objective structure, which resulted in poorly-coherent narratives such as a “Q and A style” or a “mechanical list.”

##### Non-subjective, non-objective narratives limited to outpatients

The students recognized this type of narrative had an unclear structure.

*[Until now] I had found it troublesome that I could not understand the reason why patients tended to jump from one topic to another. But when I was asked to talk as a patient and remember topics, I also talked incoherently, like an outpatient. When I was asked about my past episode, I could not remember it immediately. It took time. Then, I understood real patients’ behaviors, such as taking time to remember and remembering things suddenly.* *(Patient role, woman, Group 5; 373)*

The unclear structure of outpatients’ narratives was recognized through comparisons with SPs’ narratives.

*Student A (Doctor role, man): SPs’ description of their present illness is generally well structured.*

*Student O (Doctor role, man): That is right. I could write it up completely without additional information, from the beginning to the end.*

*Student A: It was my first time experiencing a patient talking back and forth, and complicatedly.*

*Student K (Patient role, woman): But real patients are usually incoherent.* *(Group 5; 116–122)*

#### Medical students’ recognition of content and structure of patients’ narratives: Organizing two axes and four quadrants

Patients’ narratives (as conceived by the students) were divided into four quadrants by two axes: medical versus lived content, and objective versus subjective structure. The categories were assigned quadrants as below (Fig. 3).

Quadrant I: Medical content and objective structure.
Quadrant II: Lived content and objective structure.
Quadrant III: Lived content and subjective structure.
Quadrant IV: Medical content and subjective structure.

Quadrant I included students’ recognition of narratives that could be written in medical records “as is.” This information was considered to be a necessary condition to transform outpatient narratives into disease. Quadrant II referred to students’ recognition of narratives that were possibly refutable by others, except for the medical content. In other words, Quadrant II was explained by “personal background,” which might include medical narratives about topics such as family tree, occupation, and daily life schedule. Quadrant III represented students’ recognition of narratives that patients themselves gave significance to as “lived experiences,” such as narratives about their lives and particular experiences. Quadrant IV reflected students’ recognition of narratives in which patients themselves gave significance to symptoms and suffering as “medical experiences.” According to this two-by-two grid, the students’ recognition of the characteristics of outpatient, inpatient, and SP narratives could be explained differently as follows.

First, medical students recognized that SPs’ narratives contained mostly medical content and partly lived content, and had a scientific structure. Therefore, SPs’ narratives were based on Quadrants I and II. Second, the students recognized that inpatients’ narratives were mainly based on Quadrants I and II, because their narratives acquired an objective structure by learning medical knowledge and living in hospital. Third, the students recognized that outpatients’ narratives sometimes contained Quadrants I and II, similar to those of SPs and inpatients. However, they recognized that outpatients’ narratives also contained Quadrants III and IV, because medical content, strongly based on their life, was narrated willingly. Finally, students recognized that outpatients’ narratives could be based at the middle of Quadrants I and II and Quadrants III and IV, because their narratives contained neither objective nor subjective structures.

#### Narrative irreproducibility

Medical students recognized other characteristics of outpatients’ narratives that could not be explained by the two axes above. This included how outpatients spoke differently to different medical staff (i.e., attending doctors and medical students), and even spoke differently to the same staff member on different occasions.

*Student M (Observer, woman): I imagined that she [the student “patient”] might be surprised when the outpatient answered the question from the attending doctor, although she had already tried to ask the patient [the same question, before the attending doctor asked].*

*Student T (Patient role, woman): Although I had thought that the patient had quit drinking from his comment “stopped drinking,” what I was interested in, or rather what I learned yesterday [at the ambulatory clerkship] was that patients did not always call never drinking “stopped drinking.” Because he answered “I’m still drinking” when he was asked by attending doctor whether [he was] drinking.* *(Group 7; 251, 261)*

*Patients don’t know themselves. For example, they say “I feel like this, or feel like that.” I assume patients’ answers will change by 180 degrees depending on our questions.* *(Patient role, man, Group 12; 105)*

The students compared this characteristic with inpatients’ narratives, and recognized that inpatients’ narratives did not have these characteristics, but tended to be stable with certain contents.

*Most of all, inpatients write down in notebooks. (And they explain) saying “this is it.”* *(Observer, woman, Group 6; 149–151)*

Kurtz et al. [33] noted that SPs perform a role-play scenario the same way, and described this as “reproducibility.” The students recognized that inpatients’ narratives sometimes showed a similar characteristic, as described above. In contrast, they recognized that outpatients’ narratives did not have this characteristic, and could therefore be described as having “irreproducibility.” Moreover, students also realized that outpatients’ narratives sometimes moved across different quadrants (Fig. 3).

## Discussion

We explored medical students’ recognition of the characteristics of outpatients’ narratives through an original educational program that combined outpatient encounters and subsequent medical interview training using outpatients’ narratives as peer role-play scenarios. Figure 3 describes the students’ recognition of characteristics of outpatients’ narratives using two axes (content and structure) and four quadrants. Content was determined on an axis between medical and lived, and structure on an axis between objective and subjective. The students recognized that both SPs’ and inpatients’ narratives were objectively structured with medical and lived contents, and were mainly located in Quadrants I and II. In contrast, they recognized that outpatients’ narratives extended and moved across different quadrants, which was explained as “irreproducibility.” The students recognized these characteristics through the present original educational program based on outpatient encounters and their previous interactions with SPs and inpatients.

### Considering “four quadrants of patients’ narratives” in medical communication

We conceptualized four quadrants divided by two axes (content and structure) to describe the features of patients’ narratives. Previous literature describes similar typology in patient–doctor communication. Mishler’s analysis of a series of clinical interactions revealed that doctor and patient seem to be speaking two different languages: the voice of the lifeworld and that of medicine [36]. Based on Mishler’s theory, Barry described four types of patient–doctor communication: Strictly Medicine, Mutual Lifeworld, Lifeworld Ignored, and Lifeworld Blocked [38]. The patients’ narrative that we targeted in this research can be recognized as “the voice of the lifeworld,” even though the patients’ narrative was limited in the students’ recognition. In other words, we described features of “the voice of the lifeworld” before being ignored and blocked by medical students. By recognizing those four quadrants, we can visibly explain the need to see not only quadrants I and II of patients’ narratives but also quadrants III and IV, which allows students to understand their patients’ whole world.

### Classification of patients

**Fig. 4:**
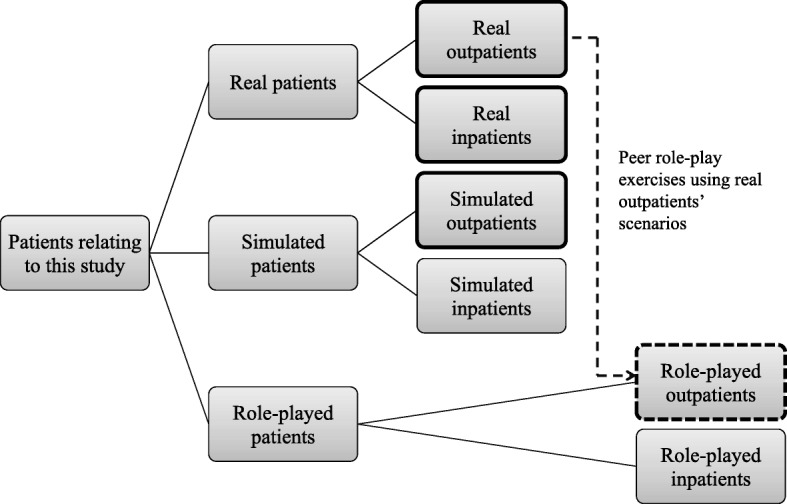
Classification of patients relating to this research. Patients circled by a bold line are those who were compared by medical students in this study. Role-played patients (circled by dotted line) reproduced generalized outpatients

In this study, participating medical students compared SPs with inpatients and outpatients. However, as real patients should be compared with SPs, we reconsidered the classification of “patients” in this study (Fig. 4) and considered SPs as “simulated outpatients” that acted as outpatients rather than as inpatients [39]. Furthermore, “role-played outpatients” (reproduced outpatients in peer role-play exercises) contributed to this educational program. As this program was based on real outpatients’ scenarios, the role-played patients reproduced outpatients. However, it is inappropriate to consider “role-played outpatients” as the reproduction of real outpatients in ambulatory practice. The narratives of “role-played outpatients” might have been inevitably biased by the students owing to their interactions with real outpatients. Therefore, these narratives may be far from the actual condition of real outpatients and we should consider “role-played outpatients” as the reproduction of generalized outpatients, without suggesting particular individuals.

It is noticeable that “role-played outpatients” in medical students’ recognition may differ, especially between students who had experienced the ambulatory practice and those that had not. Students who had experienced ambulatory practice would be able to imagine “role-played outpatients” as real outpatients. However, students who had not experienced ambulatory practice would be unable to imagine real outpatients, and would imagine patients that they had met in other educational programs. How this difference may affect medical students’ recognition of outpatients’ narratives requires further research.

### Originality of outpatients’ narratives

Medical students can recognize the differences between real patients’ and SPs’ narratives [16, 40, 41]. However, it has not previously been clarified if medical students recognize outpatients’ and inpatients’ narratives differently, even when they were both “real patients.” Outpatients’ narratives, which were the focus of this study, had characteristics of real patients’ narratives as described in previous literature [1, 16, 42]. The characteristics of inpatients’ narratives differed from those of real patients, probably because their views were overshadowed by the medical sphere. Therefore, we showed that medical students learned aspects about narrative characteristics from outpatients that would be difficult to obtain from inpatients, even if both patient groups were real patients.

### Significance of learning outpatients’ narratives in clinical clerkship

Other than in the ambulatory clerkship, real patients’ narratives are usually learned in educational programs, such as creative work about patients’ narratives, and video recordings and interviews with real patients [7, 43]. However, this is limited in the ambulatory clerkship, as real patients’ narratives are usually learned in the clerkship itself [18, 19]. Therefore, it may be argued that students do not need to learn patients’ narratives using a complicated educational program. In particular, it may not be necessary to combine the peer role-play exercise with the clerkship to learn patients’ narratives. However, learning outpatients’ narratives through the present educational program had several benefits, which would be difficult to obtain with other educational strategies.

First, this educational program offered students in their ambulatory clerkship opportunity to reflect on the perspectives of patients that they saw. In ambulatory clerkship, it is often difficult for medical students to reflect on their patients because of limitations in time, space, and teaching staff [44–46], and students may not have opportunity to reflect. However, the role-play exercise allowed students to attend to patients’ narratives. Role-play has advantages as an educational tool, such as offering students a chance to express non-verbal and emotional feelings and discuss these feelings, with group dynamics serving to stimulate students’ motivation [47]. In undergraduate medical education, role-play fosters students’ reflection of patients’ perspectives [48–50]. Therefore, combinations of learning strategies involving role-play would foster medical students’ learning about outpatients’ narratives.

Second, this educational program is part of a clinical clerkship. As students recognized in this research, real patients’ narratives naturally contain subjective structured and non-structured elements. Recognition of these structural characteristics in clinical clerkship has potential to re-stimulate medical students’ interest in patients’ narratives, which may be a tool to prevent a decline of patient-centeredness among medical students [51]. In addition, in this research suggests that medical students’ recognition of outpatients’ narratives may facilitate their understanding of the communication gap between patients and students/doctors. This means that the educational program may contribute the gradual transition from education to practice [52], particularly with respect to recognizing patients’ narratives in medical communications.

## Limitations

### Students’ comments

We interviewed students after the educational program; therefore, their comments on the program were based on recall and we might not have exactly captured their understanding about the characteristics of patients’ narratives. Ambulatory clerkship provides an appropriate opportunity to understand patients’ narratives [18, 19] and role-play fosters students’ reflection of patients’ perspectives [48–50]. In the present study, students recognized (even if only vaguely) the characteristics of outpatients’ narratives in both the ambulatory clerkship and peer role-play exercise. However, it was beyond the scope of this study to determine if students who did not participate in the focus group discussions understood the characteristics of outpatient narratives in the same way as those who participated in the focus groups. It is possible that students’ understanding was fostered by focus group discussions about patient’s perspectives after the official educational program. In other words, focus group discussion in this study had a role in students’ reflection on patients’ narratives. It may be helpful to incorporate focus groups as a part of formal education programs to foster learning about the characteristics of outpatients’ narratives.

### Peer role-play exercise

It has been argued that there is no need for students to learn patients’ narratives using a complicated educational program. This research does not suggest that peer role-play is necessary to learn patients’ narratives. Although we considered the peer role-play exercise played a significant part in the educational program, further research regarding how the peer role-play exercise influenced students’ recognition of outpatients’ narratives is necessary.

### Educational program

This study highlighted medical students’ recognition of outpatients’ narratives in the original educational program. However, this recognition was not consistent across all medical students, which might be attributable to the learning objective in the educational program. The educational program (ambulatory clerkship and peer role-play exercise) focused on students’ communication skills and clinical reasoning in the doctor role. Clinical reasoning requires students to construct a disease model from patients’ narratives [53]. Therefore, understanding outpatients’ narratives without abstracting those narratives should be considered as the learning objective in both the ambulatory clerkship and peer role-play exercise. This educational program was not sufficient to develop understanding of patients’ narratives, as it did not have such learning objectives. Correspondingly, as the educational program was considered to be insufficient for medical students to learn about patients’ illness, their recognition of outpatients’ illness did not reach a medical anthropological level.

### Sequencing other clinical clerkships

In this research, medical students compared outpatients’ narratives with those of SPs and inpatients. This highlighted a problem of sequencing other clinical clerkships. The curriculum for the current clinical clerkship program in our university is not organized based on the order in which SPs, inpatients, and outpatients are experienced in clinical clerkships. Therefore, students’ experiences were uneven with regard to their interactions with inpatients and outpatients in other clerkships. These experiences might have influenced their understanding of outpatients’ narratives.

### Research design

As this study was performed at one university in Japan, comments from participants were subject to cultural bias. However, the findings may be generalizable, as our analysis and findings were consistent with referenced knowledge from Western cultures. Furthermore, the need for an effective communication skills program to foster a smooth transition from undergraduate education to clinical practice is common among different healthcare professions, educational contexts, and countries [54, 55]. We therefore consider that our findings are generalizable to these diverse settings.

## Conclusion

We explored medical students’ recognition of the characteristics of outpatients’ narratives through an original educational program that combined outpatient encounters and subsequent medical interview training using outpatients’ narratives in peer role-play. Outpatients’ narratives as conceived by the students were divided into a two-by-two grid of four quadrants by two axes (content and structure). Outpatients’ narratives have a subjective structure, and often spread and move across different quadrants; a characteristic that can be described as “irreproducibility.” Medical students recognized these characteristics through comparisons with previous interactions with SPs and inpatients. Despite some limitations, the students learned about outpatients’ narratives in this educational program in a way that would be difficult to achieve using other educational strategies.

## Abbreviation

SPs: Simulated patients
